# Carbon Black Nanoparticles Selectively Alter Follicle-Stimulating Hormone Expression *in vitro* and *in vivo* in Female Mice

**DOI:** 10.3389/fnins.2021.780698

**Published:** 2021-12-06

**Authors:** Charlotte Avet, Emmanuel N. Paul, Ghislaine Garrel, Valérie Grange-Messent, David L’Hôte, Chantal Denoyelle, Raphaël Corre, Jean-Marie Dupret, Sophie Lanone, Jorge Boczkowski, Violaine Simon, Joëlle Cohen-Tannoudji

**Affiliations:** ^1^Université de Paris, BFA, UMR 8251, CNRS, ERL U1133, Inserm, Paris, France; ^2^Inserm U955, IMRB, U 955, Faculté de Médecine, équipe 04, Université Paris Est (UPEC), Créteil, France; ^3^Sorbonne Université, CNRS, Inserm, Neuroscience Paris Seine – Institut de Biologie Paris Seine, Paris, France; ^4^Université de Paris, BFA, UMR 8251, CNRS, Paris, France

**Keywords:** carbon black nanoparticles, pituitary, gonadotropin, GnRH, cAMP/PKA pathway, endocrine disruption

## Abstract

Toxic effects of nanoparticles on female reproductive health have been documented but the underlying mechanisms still need to be clarified. Here, we investigated the effect of carbon black nanoparticles (CB NPs) on the pituitary gonadotropins, luteinizing hormone (LH) and follicle-stimulating hormone (FSH), which are key regulators of gonadal gametogenesis and steroidogenesis. To that purpose, we subjected adult female mice to a weekly non-surgical intratracheal administration of CB NPs at an occupationally relevant dose over 4 weeks. We also analyzed the effects of CB NPs *in vitro*, using both primary cultures of pituitary cells and the LβT2 gonadotrope cell line. We report here that exposure to CB NPs does not disrupt estrous cyclicity but increases both circulating FSH levels and pituitary FSH β-subunit gene (*Fshb*) expression in female mice without altering circulating LH levels. Similarly, treatment of anterior pituitary or gonadotrope LβT2 cells with increasing concentrations of CB NPs dose-dependently up-regulates FSH but not LH gene expression or release. Moreover, CB NPs enhance the stimulatory effect of GnRH on *Fshb* expression in LβT2 cells without interfering with LH regulation. We provide evidence that CB NPs are internalized by LβT2 cells and rapidly activate the cAMP/PKA pathway. We further show that pharmacological inhibition of PKA significantly attenuates the stimulatory effect of CB NPs on *Fshb* expression. Altogether, our study demonstrates that exposure to CB NPs alters FSH but not LH expression and may thus lead to gonadotropin imbalance.

## Introduction

Reproductive processes in mammals are dependent on the appropriate regulation of the synthesis and release of luteinizing hormone (LH) and follicle-stimulating hormone (FSH) by the gonadotrope cells of the anterior pituitary. These two hormones, indeed, act in a concerted manner to regulate gonadal hormone synthesis and gametogenesis in both males and females. LH and FSH are glycoprotein hormones composed of a common α-glycoprotein subunit and distinct rate-limiting β-subunits that confer biological activity to the hormones. The level of the three subunit transcripts (*Cga*, *Lhb* and *Fshb*) as well as the release of gonadotropins are mainly controlled by the neuropeptide, gonadotropin-releasing hormone (GnRH) (Counis et al., 2005). GnRH is produced by hypothalamic neurosecretory neurons and released, in a pulsatile fashion, into the blood vessels connecting the median eminence to the anterior pituitary. Upon binding to its receptor, which is specifically expressed in gonadotrope cells, GnRH primarily activates Gq/11 proteins leading to the recruitment of a wide array of signaling pathways. GnRH notably triggers the activation of phospholipase Cβ, the mobilization of intracellular calcium and the increase of Protein Kinase C (PKC) activity, leading notably to the activation of the mitogen-activated protein kinase (MAPK) pathways (Naor and Huhtaniemi, 2013). GnRH also stimulates, indirectly or directly by coupling to Gs-proteins, the cAMP/Protein kinase A (PKA) pathway (Liu et al., 2002; Lariviere et al., 2007). All PKC, PKA and MAPK pathways contribute to the GnRH-stimulated transcription of gonadotropin genes (Thackray et al., 2010).

The balance between LH and FSH levels is finely controlled throughout reproductive life and disequilibrium in this balance is associated with reproductive disorders such as polycystic ovaries and premature ovarian failure. Polycystic ovary syndrome is characterized by an increase specifically in LH levels, whereas high levels of FSH are observed in patients or in animal models of ovarian insufficiency (Guigon et al., 2005; Vandormael-Pournin et al., 2015; Malini and Roy George, 2018). The way in which the expression of the two gonadotropins is finely and differentially regulated by GnRH is still poorly understood. GnRH differentially regulates the transcription of *Lhb* and *Fshb via* changes in pulse frequency, with increasing GnRH pulsatility favoring LH while reduced GnRH pulsatility favors FSH synthesis (Burger et al., 2004). In addition, several endocrine or locally produced factors also contribute to the fine-tuning of FSH/LH balance. Among them, is the hormone activin, which selectively activates *Fshb* transcription (Bilezikjian et al., 2012).

Reproductive fitness is extremely dependent upon environmental signals. There is growing evidence, both from wildlife and studies in human and animal models, that contaminants present in the environment affect reproductive activity (Guillette and Guillette, 1996; Danzo, 1998). Among them, industrial compounds, such as pesticides or plasticizers including phthalates and bisphenols have been identified as endocrine-disrupting chemicals that alter reproductive endocrinology and fertility. Over the past decades, a significant increase in the production and use of nanoparticles (NPs) has occurred. NPs are materials having at least one dimension less than 100 nm. Among them, carbon black NPs (CB NPs) are mainly derived from controlled incomplete combustion or thermal decomposition of hydrocarbons. A variety of engineered carbon NPs are used in consumer products such as car tires, rubber, and printer toner cartridges. Furthermore, elemental carbon-based NPs are a major part of diesel exhaust and ambient pollution (Schauer, 2003). CB NPs are mainly taken up through inhalation, and the translocation of intratracheally instilled CB NPs into blood has been described in mice (Shimada et al., 2006). Numerous experimental studies on animals have also shown that CB NP inhalation can induce pulmonary inflammation and cardiovascular diseases (Bachoual et al., 2007; Niwa et al., 2008; Bourdon et al., 2012). Further underlining their toxicity, CB NPs and their respirable aggregates/agglomerates have been classified as possibly carcinogenic to humans [group 2B] (Iyengar et al., 2016). The current limit for CB NPs exposure defined by the NIOSH (National Institute of Occupational Safety and Health) is of 3.5 mg/m^3^. Under occupational settings, however, workers could be exposed to much higher concentrations of CB NPs and levels of 79 mg/m^3^ or even 675 mg/m^3^ have been reported (IARC Working Group on the Evaluation of Carcinogenic Risks to Humans, 2010). Several recent lines of evidence suggest that CB NPs also act as endocrine-disrupting chemicals with detrimental effects on reproduction (Lu et al., 2013; Hutz et al., 2014). Intratracheal administration of CB NPs to adult male mice indeed alter testosterone production and reduces daily sperm production (Yoshida et al., 2009). Consistent with such an action, the *in vitro* exposure of a mouse Leydig cell line to CB NPs decreases the expression of the steroidogenic acute regulatory protein (StAR), which is the rate limiting factor in steroid biosynthesis (Komatsu et al., 2008). We have recently reported in human ovarian granulosa cells that CB NPs decrease basal and FSH-stimulated expression of the enzyme aromatase, which catalyzes the biosynthesis of estradiol from androgens, and also decrease estradiol secretion (Simon et al., 2017). The majority of experiments assessing the detrimental effect of environmental contaminants, and notably of NPs, on reproduction have been performed at the level of the gonads (Wang et al., 2018), and although pituitary gonadotropins are key regulators of gonadal activity, only scarce information is available on a possible disruption of pituitary endocrine activity.

The objective of this study was to investigate whether CB NPs exposure could disrupt basal or GnRH-stimulated gonadotropin secretion. To address this issue, we subjected adult female mice to non-surgical intratracheal exposure to CB NPs. To further explore the underlying mechanisms of CB NPs action, we also analyzed the effects of CB NPs *in vitro*, using two distinct models, i.e., the primary cultures of pituitary cells and the LβT2 gonadotrope cell line. We report here that CB NPs differentially alter the expression and circulating levels of LH and FSH both *in vivo* in female mice and *in vitro*. We further showed that CB NPs are internalized by gonadotrope cells and that the recruitment of the cAMP/PKA/CREB pathway by CB NPs mediates the increase in FSH expression. Altogether, our study suggests that CB NPs may act as an endocrine disruptor, leading to an imbalance of the gonadotropins, LH and FSH.

## Materials and Methods

### Exposure of Female Mice to Carbon Black Nanoparticles

The CB NPs used were FW2 (13 nm) and obtained from Evonik Industries/Degussa (Frankfurt, Germany). Their key physico-chemical characteristics including diameter, surface area, zeta potentials and hydrodynamics of the suspended particles, have been determined previously (Sanfins et al., 2011). Stock solutions of 20 mg/mL of CB NPs were prepared and sonicated as previously described (Simon et al., 2017).

Studies were conducted on twelve-week-old adult female C57BL/6 mice. Mice were maintained under controlled conditions (12-h light/dark cycle) with food and water available *ad libitum*. Once a week over four weeks, female mice received 10 μL of vehicle (NaCl group, 30 animals) or CB NPs at 10 mg/mL diluted in NaCl (CB NP group, 36 animals) that were administered by non-surgical intratracheal instillation performed under anesthesia [1.6 mg ketamine (Virbac, Carros, France) and 300 mg xylazine (Bayer^TM^, Puteaux, France)]. The protocol has been approved by our local ethic committee (ComEth Anses/ENVA/UPEC) under the reference #12-104 (final approval #20/12/12-27). Intratracheal exposure to 100 μg CB NPs corresponds to the mice being exposed at the current occupational exposure limit of 3.5 mg/m^3^ for 5,5 working days (8 h/day) as previously reported (Bourdon et al., 2013; Husain et al., 2015). Estrous cyclicity was monitored during the 12 days preceding the first exposure to CB NPs or NaCl and the 12 days preceding sacrifice. Vaginal cells from females were collected by daily saline wash and analyzed after May-Grünwald-Giemsa R (RAL Diagnostics) staining. Stages of the estrous cycle were characterized, as previously described (Vandormael-Pournin et al., 2015). Mice were sacrificed two weeks after the last exposure. Retro-orbital blood, anterior pituitary glands, lungs and ovaries were collected. Serum was separated from blood by centrifuging 10 min at 1,000 × *g* and stored at −20°C. Body and organs weights were measured. Anterior pituitaries, lungs and ovaries were deep-frozen in liquid nitrogen and stored at −80°C. Four mice died during the course of the experiment (3 NaCl- and 1 CB NP-treated mice). Moreover, because of insufficient RNA quantity or quality, pituitary gonadotrope function was eventually measured on 24 NaCl- and 33 CB NP-treated mice.

### Cell Culture and Exposure to Carbon Black Nanoparticles

The pituitary gonadotrope LβT2 cell line was provided by Dr. Pamela Mellon (University of California, San Diego) (Thomas et al., 1996; Turgeon et al., 1996) and maintained in 12-well plates (1 × 10^6^ per well) in DMEM (Gibco, Life Technologies) supplemented with 10% fetal bovine serum (FBS) and 0.5% Penicillin/Streptomycin (P/S) (Sigma-Aldrich). Primary cultures of anterior pituitary cells were prepared from adult female Wistar rats (225–250 g, Janvier) as previously described (Garrel et al., 2010) and cultured in 12-well plates or 6-well plates (1 × 10^6^ per well) for 48 h in Ham F-10 medium with 10% FBS and 0.5% P/S. Before exposure to CB NPs, cells were starved overnight in serum-free medium and then incubated in serum-free medium for 24 h with increasing concentrations of CB NPs (25–100 μg/mL corresponding to 5–20 μg/cm^2^, as previously done on human luteinized granulosa cells and on the granulosa cell line KGN (Simon et al., 2017). These concentrations are in the range or even lower than those classically used in several other *in vitro* studies (Yamawaki and Iwai, 2006; Komatsu et al., 2008; Lee et al., 2012). At the end of incubation, medium was collected for assaying gonadotropin secretion. Cells were extensively washed with culture medium and total RNAs were isolated. Shorter exposures (30 or 60 min) to CB NPs were performed to analyze CB NP activation of cellular signaling pathways. The potent and selective PKA inhibitor Rp-cAMP (100 μM) was added 30 min before CB NP addition. LβT2 cells were also stimulated during 6 h with the GnRH agonist Triptorelin (GnRHa, 100 nM) after having been exposed for 24 h to increasing concentrations (25–100 μg/mL) of CB NPs.

### Cell Viability

The viability of cultured anterior pituitary and LβT2 cells exposed to CB NPs was measured using the MTT (3-(4,5-dimethylthiazole-2-yl)-2,5-diphenyltetrazolium bromide) assay. Cells were serum-starved overnight and incubated for 24 h with increasing concentrations of CB NPs (25–100 μg/mL corresponding to 5–20 μg/cm^2^). Media were then aspirated and replaced by 0.5 mL medium containing MTT (1 mg/mL). After 2 h of incubation, cells were lysed and treated with 200 μL of dimethyl sulfoxide (DMSO). The absorbance of the solubilized formazan crystals was read at 575 nm on a Flexstation3 (Molecular devices).

### Transmission Electron Microscopy

LβT2 cells seeded at 2 × 10^6^ cells per well in 6-well plates in 2 mL of DMEM containing 10% FBS and 0.5% P/S and confluent primary cultures of anterior pituitary cells cultured in 6-well plates in 2 mL of Ham F-10 medium with 10% FBS and 0.5% P/S, were used. Forty-eight hours later, cells were serum starved overnight and incubated or not the next day with CB NPs (LβT2: 10 or 25 μg/mL; primary cultures: 50 μg/mL) for 24 h. Cells were then extensively washed with culture medium and washed in 0.05 M sodium cacodylate buffer (pH 7.4) before being fixed *in situ* with 2.5% glutaraldehyde diluted in the same buffer at 4°C for 1 h. After five washes in sodium cacodylate buffer, cell monolayers were post-fixed for 1 h using 2% osmium tetroxide in sodium cacodylate buffer in the dark at room temperature. Dehydration *in situ* was performed through an ethanol ascending series and cell monolayers were embedded in epoxy resin (Epoxy-Embedding Kit, cat. # 45359, Sigma Aldrich, Switzerland). Gelatin capsules filled with resin were returned to the cell layers and the polymerization proceeded in a 60°C oven for 48 h. Capsules were then detached from the wells and ultrathin sections (70 nm) of the cell monolayer were collected on copper grids. Sections were contrasted using 2% uranyl acetate for 5 min and Reynold’s lead citrate for 2 min. Examination was done using a transmission electron microscope (80–120 kV 912 Omega ZEISS) equipped with a digital camera (Veleta Olympus).

### Hormone Level Assays

LH and FSH were simultaneously assayed in 10 μL of serum with the Luminex technology using the mouse pituitary magnetic bead panel Milliplex Map kit (Merck-Millipore, Nottingham, United Kingdom) in accordance with the manufacturer’s instructions. Serum levels of inhibin B were measured using a commercially available ELISA kit (Beckman Coulter) following manufacturer’s protocol. The concentration of estradiol and progesterone were assayed in the serum using gas chromatography coupled with mass spectrometry (GC-MS) procedure, as described previously (Giton et al., 2015; Francois et al., 2017). Due to insufficient serum volume for this analysis, serum sex steroid levels were measured in a smaller subset of females than used for gonadotropin assays (16 NaCl- and 24 CB NP-treated mice for progesterone and 11 NaCl- and 18 CB NP-treated mice for estradiol). The linearity of steroid measurement was confirmed by plotting the ratio of the respective steroid peak response/internal standard peak response to the concentration used for calibration standard. Lower limit of quantification was 0.2 pg for estradiol and 5.5 pg for progesterone. In cell culture media, LH and FSH concentrations were measured using an ELISA method adapted from Faure et al. (2005) with reagents supplied by Dr. Parlow (NHPP, Harbor-UCLA, CA, United States) as previously described (Garrel et al., 2011; Lannes et al., 2016). The minimum detectable LH and FSH concentrations were 0.2 and 1 ng/mL, respectively and interassay coefficients of variation were less than 10%. We checked that CB NPs present in the cell culture medium did not interfere with the binding between the antibody and the FSH or LH standards in the ELISA assay (data not shown).

### Reverse-Transcription and Real-Time PCR

Total RNAs from LβT2 and cultured pituitary cells as well as from mouse anterior pituitaries and lungs were isolated with a RNeasy-kit (Qiagen, France). Reverse transcription (RT) was performed using 1 μg RNA in a total volume of 20 μL with the Reverse transcription using superscript II reverse transcriptase (Invitrogen) and real-time PCR were carried out in the LightCycler 480 Instrument (Roche Diagnostics) as previously described (Simon et al., 2017). Gene expression levels were normalized to *Hprt*, encoding hypoxanthine phosphoribosyltransferase, for mouse anterior pituitaries, to *Sf3a1* encoding Splicing factor 3 subunit 1 for mouse lungs and to *Cyclophillin* for LβT2 and cultured pituitary cells. The oligonucleotide primer sequences are indicated in Table 1. Primers were designed to target both rat and mouse DNA sequences. Data were analyzed using the Advanced-E-method with standard-curve derived efficiencies obtained from LightCycler 480 software. The specificity of amplification was checked by gel electrophoresis and melting curve analysis.

**TABLE 1:** Oligonucleotide primer sequences used for real-time PCR performed on primary anterior pituitary, LβT2 cells or lung. *Fshb*, *Lhb*, *Cga*, *Fst*, *Fs-288, Hprt*, *Cyclophilin*, *Tnfa*, *Il1b*, *Il6* and *Sf3a1*.

**Target**	**Forward primer**	**Reverse primer**
*Fshb*	5′ TTGCATCCTACTCTGGT GCT 3′	5′ AGCTGGGTCCTTATACA CCA 3′
*Lhb*	5′ ATCACCTTCACCACCAG CAT 3′	5′ GACCCCCACAGTCAGAG CTA 3′
*Cga*	5′ GCTGTCATTCTGGTCAT GCT 3′	5′ GAAGCAACAGCCCATAC ACT 3′
*Fst*	5′ CAAGGTTGGCAGAGGTC GCT 3′	5′ CCGAGATGGAGTTGCAA GAT 3′
*Fs-288*	5′ CTCTCTCTGCGATGAGC TGTGT 3′	5′ GGCTCAGGTTTTACAGGC AGAT 3′
*Cyclophilin*	5′ CAAAGTTCCAAAGACAG CAG 3′	5′ CTGGCACATGAATCCTG GAA 3′
*Hprt*	5′ AGGACCTCTCGAA GTGT 3′	5′ ATTCAAATCCCTGAAGTA CTCAT 3′
*Tnfa*	5′ CCACCACGCTCTTCTGTC TACTGAACTT 3′	5′ GTGGGCTACAGGCTTGTC ACTCG 3′
*Il1b*	5′ TGAGAATGACCTGTTCTT TGAAGTTG 3′	5′ GACAGCCCAGGTCAAAG GTTT 3′
*Il6*	5′ TGAATTGGATGGTCTTGGT CCTTAGCCAC 3′	5′ ACAAAGCCAGAGTCCTTCA GAGAGATACAG 3′
*Sf3a1*	5′ CCACTGAGTCCAAACAGC CAAT 3′	5′ AGCTTCAAATTCAGGC CCAT 3′

### Protein Extraction and Immunoblotting

Membrane proteins were prepared from LβT2 cells as previously described (Garrel et al., 2016). Equal amounts of protein (20 μg) were separated on a 10% SDS-PAGE and transferred to a nitrocellulose membrane. Specific antibodies and Pierce ECL2 substrate were used to detect Phospho-CREB (P-CREB; Cell signaling #9198; 1:1000), Phospho-Extracellular signal-regulated kinase1/2 (P-ERK 1/2; Cell signaling #9101; 1:1000), Phospho-p38 (P-p38; Cell signaling #4511; 1:1000), Phospho-Jun-kinase (P-JNK; Cell signaling #4671; 1:1000), Phospho-Smad2 (P-Smad2; Cell signaling #3108; 1:1000). Respective non-phosphorylated proteins, including Total CREB (Cell signaling #9197), Total ERK1/2 (Cell signaling #9102), Total p38 (Cell signaling #8690), Total JNK (Cell signaling #4671), Total Smad2 (Cell signaling #5339) were also detected (dilution of antibodies, 1:1000) as well as vinculin (Sigma-Aldrich #V9131; 1:20 000), used as an internal loading control. Blots were analyzed with a Fuji LAS-4000 imager and quantified using MultiGauje software. Full scans of the entire original gels are presented in Supplementary Materials 1, 2.

### Statistical Analyses

Data are from at least three independent experiments. The precise number is indicated in the legends of figures. All data were analyzed using the Prism 6 Software (GraphPad Software, Inc). Data were first subjected to normality test and data that did not pass the test were analyzed using non-parametric tests Mann-Whitney or Kruskal-Wallis followed by Dunnet’s multiple comparison test. *P* ≤ 0.05 was considered as significant.

## Results

### *In vivo* Exposure to Carbon Black Nanoparticles Disrupts Pituitary Gonadotrope Activity and Selectively Increases Follicle-Stimulating Hormone in Female Mice

**FIGURE 1:**
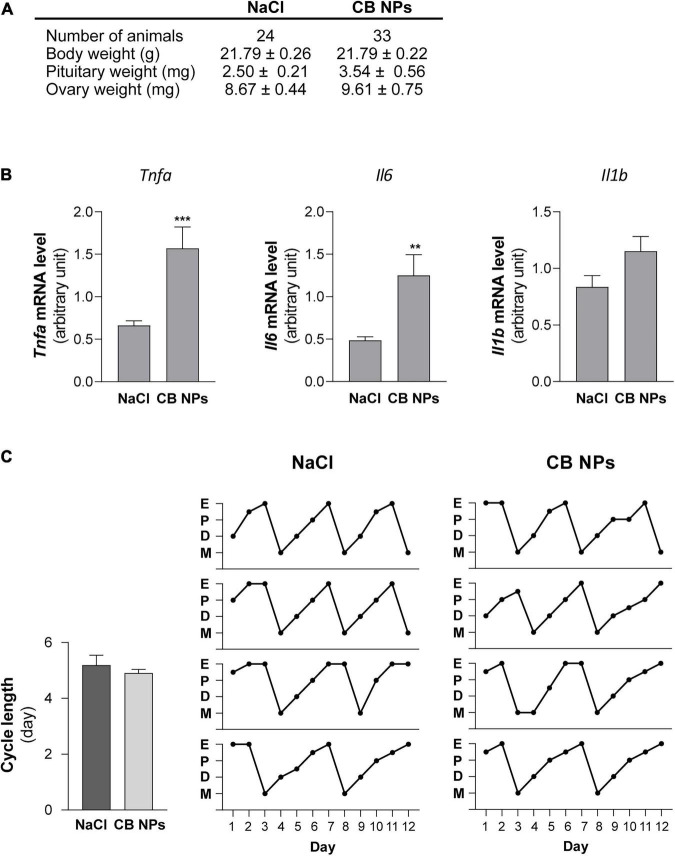
Impact of CB NPs exposure on mice body and organs weights, pulmonary pro-inflammatory cytokine transcripts and estrous cyclicity. **(A)** Effect of CB NPs on body weights and relative organ weights of female mice after an intratracheal instillation with 100 μg CB NPs/week during 4 weeks. **(B)** Transcript levels of canonical pro-inflammatory cytokines in lungs of female mice exposed to CB NPs. Control saline solution (NaCl, 0.9%) or CB NPs (100 μg) were intratracheally administered to C57BL/6 mice once a week during four weeks as described in the Materials and Methods. Mice were sacrificed and lungs were collected. *Tnfa*, *Il6* and *Il1b* mRNA levels were determined by real-time quantitative PCR in NaCl- and CB NP-treated mice (11 animals in each group). **(C)** Effect of CB NPs on mice estrous cyclicity. Left, Estrous cycle lengths were determined following CB NP exposure during the 12 days preceding sacrifice. No difference in cycle lengths was observed between mice exposed to NaCl or CB NPs (5.19 ± 0.35 in control mice (*n* = 24) versus 4.91 ± 0.13 days in CB NP-treated mice (*n* = 33). Right, Diagrams representing the estrous cycles at each stage (E: estrous; P: proestrous; D: diestrous; M: metestrous) for four representative controls and CB NPs mice monitored daily during the 12 days preceding sacrifice. At the time of sacrifice, percentages of mice in each stage of the estrous cycle were roughly similar between the two groups of mice: 16.7, 25, 29.1, and 29.2% in proestrous, estrous, diestrous and metestrous, respectively in controls vs. 18.2, 30.3, 24.2, and 27.3% in proestrous, estrous, diestrous and metestrous, respectively in CB NP-treated mice. Data are expressed as means ± SEM. Statistical differences were determined by the Mann-Whitney test. **p < 0.01 and ***p < 0.001 compared to NaCl.

**FIGURE 2:**
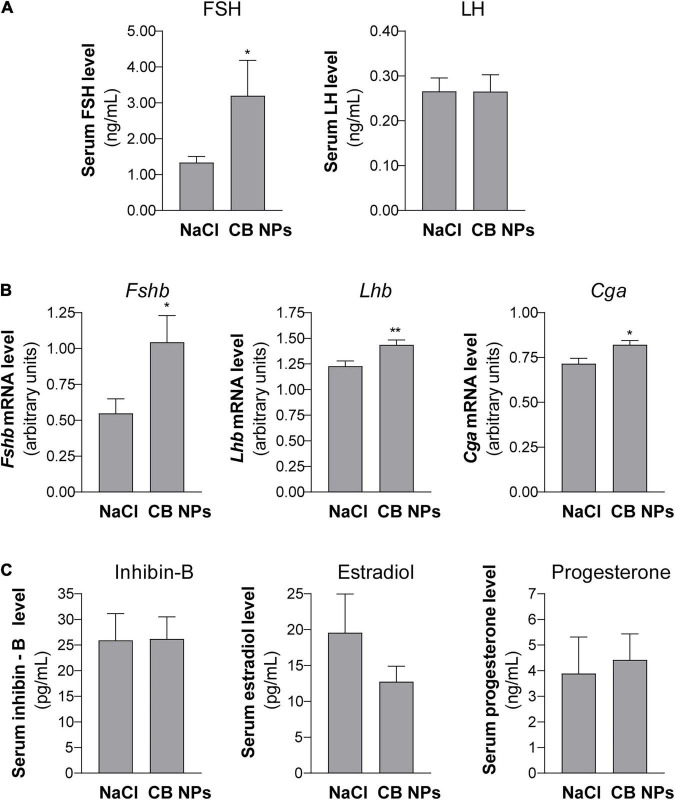
Pituitary gonadotropin serum levels and subunit gene expression in female mice exposed to CB NPs. CB NPs (100 μg) or control saline solution (NaCl, 0.9%) were intratracheally administered to C57BL/6 mice once a week during four weeks as described in the Materials and Methods. Mice were sacrificed and anterior pituitaries and blood were collected. Concentrations of FSH and LH were determined in serum by Luminex technology in 24 NaCl- and 33 CB NP-treated mice **(A)**. *Fshb*, *Lhb* and *Cga* mRNA levels were determined by real-time quantitative PCR in 24 NaCl- and 33 CB NP-treated mice **(B)**. Serum levels of inhibin B and sex steroids (estradiol and progesterone) were determined by ELISA and GC-MS, respectively, as described in the materials and methods **(C)**. Assays were performed on 23 NaCl- and 30 CB NP-treated mice for inhibin-B, 11 NaCl- and 18 CB NP-treated mice for estradiol and 16 NaCl- and 24 CB NP-treated mice for progesterone. Data are represented as mean ± SEM. Statistical differences were determined by the Unpaired *t*-test (for B, *Fshb* mRNA) or Mann-Whitney test. *p < 0.05 and **p < 0.01 compared to NaCl.

Exposure of female mice to CB NPs was carried out by non-surgical intratracheal instillations of CB NPs performed once weekly over four weeks. Mice did not exhibit any obvious signs of distress (loss of activity, mobility difficulties, respiratory distress, bristly fur or arched back) during the treatment period. Control mice were treated with NaCl. Body weight as well as pituitary and ovarian weights were unaffected by the treatment (Figure 1A). As previously reported (Roulet et al., 2012), intratracheal CB NP instillations led to pulmonary inflammation, as revealed by a significant increase in the pulmonary expression of pro-inflammatory cytokines such as tumor necrosis factor (TNF) α and interleukin 6 (IL-6) in female mice (Figure 1B). Examination of vaginal cytology showed the presence of all stages of the estrous cycle in both groups of mice. Daily analyses of vaginal smears over twelve days (approximately three consecutive cycles) are illustrated for 4 CB NP and 4 control females in Figure 1C, and revealed similar patterns of estrous cyclicity as well as similar cycle lengths in control and CB NP-treated mice [5.19 ± 0.35 in NaCI- (*n* = 24) *versus* 4.91 ± 0.13 days in CB NP-treated mice (n = 33)] (Figure 1C). To determine whether CB NPs could disrupt pituitary gonadotrope activity, we measured serum concentrations of gonadotropins as well as gonadotropin subunit transcript levels in both NaCl (control)- and CB NP-treated mice. Exposure to CB NPs significantly increased circulating FSH as compared to controls (2.7 ± 0.8-fold, *p* < 0.05, Figure 2A). Interestingly, no significant change in circulating LH could be detected. The treatment of mice with CB NPs also led to a two-fold elevation in the level of pituitary *Fshb* transcripts compared to levels in control mice (2 ± 0.3-fold, *p* < 0.05, Figure 2B). In contrast, albeit significant, the elevation was much more modest for *Lhb* and *Cga* transcripts (Figure 2B). We then investigated whether changes in ovarian hormones could account for the observed changes in gonadotropin expression. Inhibin produced by granulosa cells of growing ovarian follicles is known to target the pituitary and antagonize activin, leading to a selective decrease in *Fshb* transcription and release (Makanji et al., 2014). However, as illustrated in Figure 2C, no difference in serum levels of inhibin was observed between NaCl- and CB NP-treated mice. We also measured the sex steroids, estradiol and progesterone, by GC-MS analysis, which is the most accurate and reliable method to measure steroids. As observed for inhibin, serum progesterone level displayed no significant changes following CB NP treatment. This was also the case for serum estradiol levels, although a tendency to decrease upon CB NP treatment could be observed.

### *In vitro* Exposure of Anterior Pituitary Cells to Carbon Black Nanoparticles Alters Follicle-Stimulating Hormone but Not Luteinizing Hormone Expression and Secretion

**FIGURE 3:**
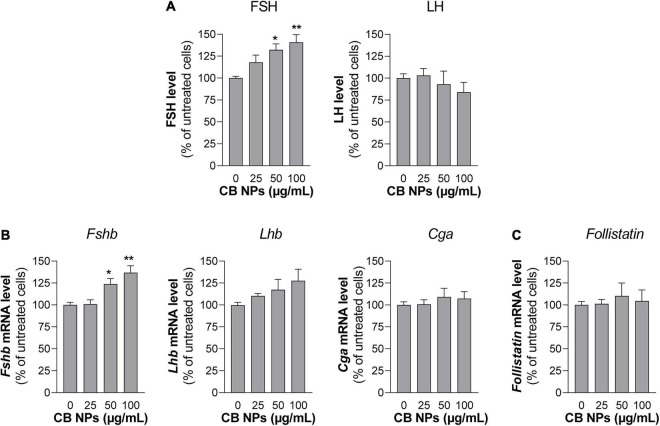
Gonadotropin secretion and gene expression in primary cultures of anterior pituitary cells treated with CB NPs. Rat primary anterior pituitary cells were cultured for 24 h with increasing concentrations of CB NPs (25–100 μg/mL). At the end of the incubation, FSH and LH concentrations in the culture media of primary pituitary cells were determined by ELISA. FSH/LH ratio is also shown in panel **(A)**. Total RNAs were extracted and *Fshb*, *Lhb*, *Cga* **(B)** and *Follistatin* **(C)** mRNA levels were determined by real-time quantitative PCR. Data are expressed as percentage over untreated cells and expressed as means ± SEM from 4 independent experiments. Statistical differences were determined by non-parametric Kruskal-Wallis test followed by Dunnet’s multiple comparison test. *p < 0.05 and **p < 0.01 compared to no CB NPs.

**FIGURE 4:**
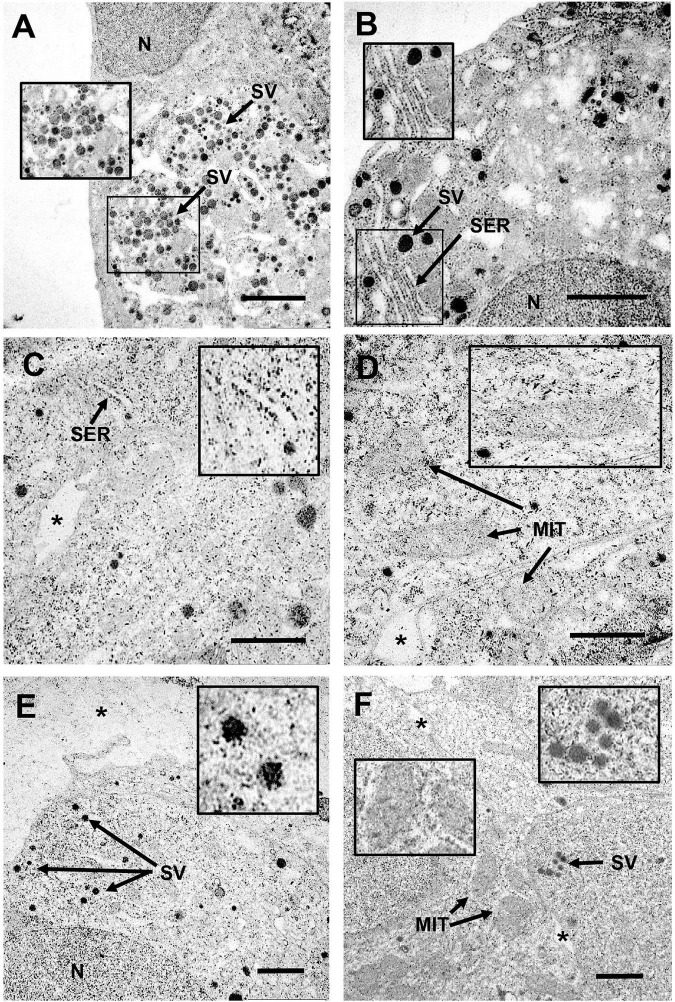
Transmission electron microscopy study of uptake and intracellular localization of CB NPs in primary cultures of anterior pituitary cells and in LβT2 cells. Primary cultures of rat anterior pituitary cells were treated for 24 h with CB NPs at a concentration of 50 μg/mL **(A,B)**. LβT2 gonadotrope cells were incubated for 24 h with **(C–E)** or without **(F)** CB NPs at a concentration of 25 μg/mL. Cells were then extensively washed and processed for transmission electron microscopy as described in Materials and Methods. In CB NP-treated cells **(A–E)**, aggregates/agglomerates of CB NPs were freely dispersed within the cytoplasm or associated with different subcellular compartments and organelles in primary pituitary cells **(A,B)** and in LβT2 cells **(C–E)**. Arrows indicate mitochondria (MIT), secretory vesicles (SV) and smooth endoplasmic reticulum (SER). Magnifications illustrate the close association of CB NPs with smooth endoplasmic reticulum **(B,C)**, mitochondria **(D)** or secretory vesicles **(A,E)**. In contrast, no CB NPs could be detected in control untreated primary cultures (data not shown) and LβT2 cells **(F)**. Note that almost no CB NPs aggregates/agglomerates could be detected in the bottom of the culture dish or in intercellular spaces as compared to intracellular compartment (*). *N*, nucleus. Scale bar: 2 μm.

To determine whether CB NPs could directly act on the pituitary to disrupt gonadotrope activity, we first treated primary cultures of anterior pituitary cells with increasing concentrations of CB NPs (25–100 μg/mL) for 24 h. The potential cytotoxic effect of CB NPs was determined by measuring cell viability with the commonly used MTT assay. We have previously shown that the presence of CB NPs does not interfere with this assay (Simon et al., 2017). As indicated in Table 2, the incubation of cells over 24 h with 50 or 100 μg/mL CB NPs did not alter cell viability. Treatment with CB NPs induced a dose-dependent increase in FSH secretion, as shown in Figure 3A. The increase was significant from 50 μg/mL, with a maximum increase of 141 ± 9% as compared to untreated cells being observed at 100 μg/mL. In contrast, CB NPs did not affect LH secretion whatever the concentration used (Figure 3A). We next determined the effects of CB NPs on the transcript levels of gonadotropin subunits by real-time PCR (Figure 3B). CB NPs significantly and dose-dependently increased *Fshb* transcript levels with an increase of 137 ± 8% at 100 μg/mL CB NPs. No significant change in *Lhb* or *Cga* transcript levels could be observed after treatment with CB NPs. Because follistatin, produced by gonadotrope and folliculo-stellate cells of the pituitary, binds to activin and antagonizes its action (Bilezikjian et al., 2012), we also measured follistatin transcript levels which were unaffected by the treatment of anterior pituitary cells with increasing concentrations of CB NPs (Figure 3C). Alternative splicing of follistatin mRNA can result in the formation of a shorter 288 amino acid isoform (FS-288), which was reported to be more active in suppressing FSH release by rat pituitary cell cultures (Sugino et al., 1993). As observed for total follistatin, the transcript levels of the active FS-288, determined using specific primers (Boerboom et al., 2015; Table 1), were not affected by the treatment with CB NPs (123 ± 11% and 118 ± 16% of the levels in control cells with 50 and 100 μg/mL of CB NPs, respectively, data not shown). Transmission electron microscopic analyses were performed on primary cultures of pituitary cells (Figures 4A,B). Aggregates/agglomerates of CB NPs were visualized in all observed cells exposed to 50μg/mL of CB NPs. They were freely dispersed in the cytoplasm or associated with different subcellular compartments such as the smooth endoplasmic reticulum and electron-dense vesicles. These dense-core vesicles, of a diameter of approximately 150 nm, which were abundantly present in the cytoplasm, correspond to secretory vesicles (Figures 4A,B).

**TABLE 2:** Effect of CB NPs on cellular viability of LβT2 cells or primary cultures of anterior pituitary cells.

**CB NPs (μg/mL)**	**0**	**25**	**50**	**75**	**100**
LβT2	100	99 ± 2	101 ± 3	101 ± 2	97 ± 1
Pituitary primary culture	100	–	107 ± 3	–	106 ± 2

*LβT2 cells and primary cultures of anterior pituitary cells were treated with increasing concentrations of CB NPs (25–100 μg/mL) for 24 h and the MTT-based colorimetric assay was used to quantify cell viability. Absorbance was read at 575 nm and data are expressed as percentage over untreated cells and are the mean ± SEM from 4 independent experiments. Statistical differences were determined by non-parametric Kruskal-Wallis test followed by Dunnet’s multiple comparison test. Data showed no statistical significance.*

### Carbon Black Nanoparticles Enter LβT2 Gonadotrope Cells and Selectively Increase *Fshb* Transcript Levels

**FIGURE 5:**
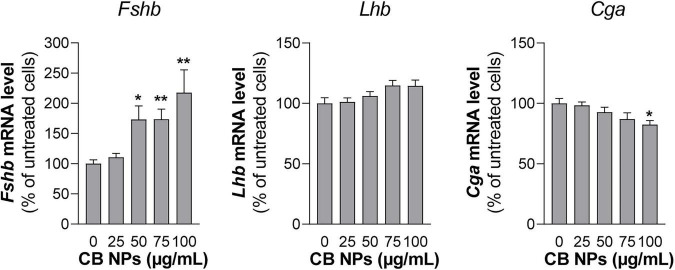
Gonadotropin gene expression in LβT2 gonadotrope cells treated with CB NPs. LβT2 gonadotrope cells were cultured for 24 h with increasing concentrations of CB NPs (25–100 μg/mL). At the end of the incubation, cells were extensively washed, total RNA were extracted and *Fshb*, *Lhb* and *Cga* mRNA levels were determined by real-time quantitative PCR. Data are expressed as percentage over untreated cells and expressed as means ± SEM from 5 to 8 independent experiments. Statistical differences were determined by non-parametric Kruskal-Wallis test followed by Dunnet’s multiple comparison test. *p < 0.05 and **p < 0.01 compared to no CB NPs.

To further determine the effects of CB NPs on gonadotropin expression, we used the most differentiated gonadotrope cell line available: the LβT2 gonadotrope cell line (Pernasetti et al., 2001). As observed with primary cultures of pituitary cells, the incubation of LβT2 cells for 24 h with CB NPs, at concentrations up to 100 μg/mL did not have any effect on cell viability (Table 2). We next determined the uptake and intracellular localization of CB NPs by transmission electron microscopy analysis using two concentrations of CB NPs, 10 μg/mL (not illustrated) and 25 μg/mL (Figures 4C–E). CB NPs were observed in the cytoplasm as freely dispersed aggregates/agglomerates within or associated with different subcellular compartments or organelles such as the smooth endoplasmic reticulum (Figure 4C) and mitochondrial membranes (Figure 4D). CB NPs were also found within the numerous electron-dense vesicles (Figure 4E), corresponding to secretory granules. In contrast and as expected, no CB NPs could be detected in non-exposed cells (Figure 4F). We next determined the effects of CB NPs on gonadotropin expression in the LβT2 cell line. As observed with primary cultures of pituitary cells, treatment with CB NPs significantly and dose-dependently increased *Fshb* expression (maximum increase of 218 ± 38% at 100 μg/mL). No effect could be detected on *Lhb* transcript levels while a small decrease in *Cga* transcript levels was observed at 100 μg/mL of CB NPs (Figure 5).

### Carbon Black Nanoparticles Increase *Fshb* Expression Through the cAMP Pathway in LβT2 Cells

**FIGURE 6:**
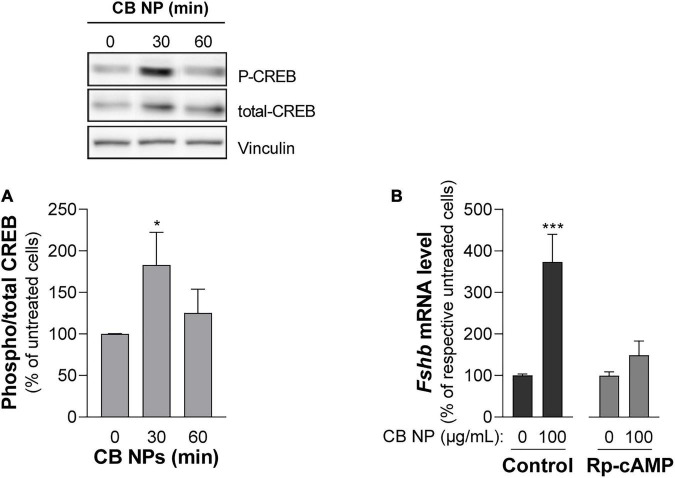
Role of the cAMP pathway in the effects of CB NPs on *Fshb* expression in LβT2 cells. **(A)** LβT2 cells were cultured for the indicated time with 100 μg/mL of CB NPs. Phospho-CREB and total CREB protein levels were analyzed by immunoblotting. Phospho-CREB protein levels were normalized by total CREB signals (histogram) and expressed as percentage over untreated cells. There was no significant alteration of total CREB content after CB NP treatment as assessed by normalization with vinculin (total CREB/vinculin ratio was of 145 ± 21% and 142 ± 20% of control at 30 and 60 min, respectively, *p* > 0.05). Data are the mean ± SEM of 4 or 5 independent experiments. Statistical differences were determined by non-parametric Kruskal-Wallis test followed by Dunnet’s multiple comparison test. *p < 0.05 compared to no CB NPs. **(B)** LβT2 cells were cultured for 24 h with or without 100 μg/mL of CB NPs and PKA inhibitor, Rp-cAMP (100 μM). Total RNAs were extracted and *Fshb* mRNA levels were determined by real-time quantitative PCR. Data are expressed as percentage over control untreated cells (0 CB NPs) and are the mean ± SEM of 8 independent experiments. Basal *Fshb* mRNA levels were not significantly affected by treatment with the PKA inhibitors. Statistical differences were determined by the Mann-Whitney test. ***p < 0.001 compared to respective untreated cells.

**FIGURE 7:**
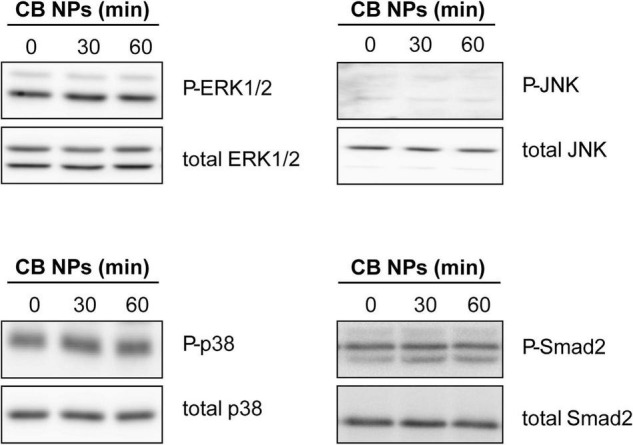
Analysis of MAPK and Smad2 signaling pathways in LβT2 cells treated with CB NPs. LβT2 cells were cultured for the indicated times with 100 μg/mL of CB NPs. Total and phosphorylated Erk1/2, p38, JNK and Smad2 were analyzed by immunoblotting as indicated in Materials and Methods. Seven independent experiments were performed and a representative immunoblot is shown for each protein.

We next conducted experiments in LβT2 cells in order to understand the mechanisms of action of CB NPs. Several signaling pathways can be activated following cell exposure to carbon NPs (Brown et al., 2000; Sydlik et al., 2006; Simon et al., 2017; Cheng et al., 2019). Recruitment of PKA and PKA-dependent phosphorylation of the cAMP response element-binding protein (CREB) transcription factor have been identified as key mechanisms mediating the preferential activation of *Fshb* transcription by GnRH in LβT2 cells (Thompson et al., 2013). We thus first sought to determine whether the PKA/CREB pathway could be recruited by CB NPs by measuring the phosphorylation of the PKA related transcription factor CREB. LβT2 cells were stimulated for 30 or 60 min with 100 μg/mL of CB NPs and CREB phosphorylation measured by immunoblotting with antibodies recognizing the phosphorylated and total forms of CREB. Treatment of cells with CB NPs for 30 min enhanced the phosphorylation of CREB as compared to control conditions (Figure 6A inset), significantly increasing the ratio of phosphorylated to total CREB (by 183 ± 39%, *p* < 0.05; Figure 6A). To further investigate the role of the cAMP/PKA signaling pathway in mediating CB NP-stimulated *Fshb* expression, we treated LβT2 cells with CB NPs in the presence of a selective pharmacological inhibitor of PKA, Rp-cAMP (Figure 6B). As expected, CB NP treatment significantly increased *Fshb* transcript level (by 374 ± 66%, *p* < 0.001) in control cells. Pharmacological inhibition of PKA significantly reduced the CB NP-dependent increase in *Fshb* transcript levels, suggesting that this increase was mediated at least in part by PKA. Because CB NPs have been reported to activate MAPK pathways in several cell lines, including reproduction-linked cell lines such as the ovarian granulosa cells (Sydlik et al., 2006; Simon et al., 2017), we also examined the ability of CB NPs to activate the three branches of the MAPK pathway: ERK1/2, JNK and p38 (Figure 7). Detection of phosphorylated and total forms of these kinases was carried out after 30 and 60 min of treatment with CB NPs, as done for CREB. Immunoblotting analysis revealed that, at least during the time period studied, CB NPs did not activate MAPK signaling pathways as no increase of phosphorylation of ERK1/2, p38 or JNK could be detected (P-ERK1/2/total ERK1/2: 115 ± 11% and 113 ± 10% of control levels after 30 and 60 min of CB NP treatment, respectively; P-p38/total p38: 107 ± 25% and 92 ± 18% of control levels after 30 and 60 min of CB NP treatment, respectively; P-JNK/total JNK: non-quantifiable). In contrast, and as expected, a GnRH agonist (GnRHa: triptorelin, 100 nM) increased their phosphorylation levels (not shown). Because the Smad2/3 pathway strongly stimulates *Fshb* transcription (Bernard, 2004; Thackray et al., 2010), we further examined the ability of CB NPs to activate this pathway in LβT2 cells by immunodetection of the phosphorylated form of Smad2. As illustrated in Figure 7, CB NPs did not increase Smad2 phosphorylation, suggesting that this pathway is not recruited by CB NPs in LβT2 cells. Under these experimental conditions, Smad2 phosphorylation could be induced by activin A (data not shown).

### Carbon Black Nanoparticles Increase Gonadotropin-Releasing Hormone Stimulation of *Fshb* but Not *Lhb* Expression in LβT2 Cells

**FIGURE 8:**
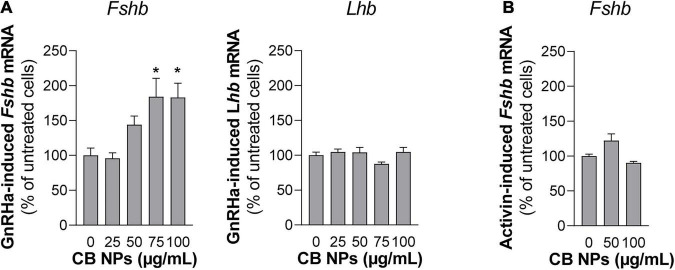
Effects of CB NPs on GnRH agonist stimulation of *Fshb* and *Lhb* expression in LβT2 cells. LβT2 cells were cultured for 24 h with increasing concentrations (25–100 μg/mL) of CB NPs, washed and incubated or not for an additional 6 h with the GnRH agonist Triptorelin [GnRHa, 100 nM; **(A)**] or Activin A [10 ng/mL; **(B)**]. Total RNAs were extracted, and Fshb and Lhb mRNA levels were determined by real-time quantitative PCR as indicated in Materials and Methods. Data are expressed as percentage over GnRHa **(A)** or Activin A **(B)** stimulation in absence of CB NPs and are the mean ± SEM from 3 to 6 independent experiments. Statistical differences were determined by non-parametric Kruskal-Wallis test followed by Dunnet’s multiple comparison test. *p < 0.05 compared to no CB NPs.

Since GnRH is the key regulator of gonadotrope cell activity, we next examined whether CB NPs could alter the GnRH-dependent-induction of gonadotropin synthesis. LβT2 cells were pre-treated with increasing concentrations of CB NPs for 24 h followed by a 6 h-treatment with the GnRH agonist (Figure 8A). As expected, GnRHa significantly increased *Fshb* and *Lhb* transcript levels compared to controls (182 ± 19% and 130 ± 6%, respectively, *p* < 0.05). Treatment of cells with CB NPs significantly amplified the GnRH-dependent stimulation of *Fshb* transcript levels. Maximal amplification was attained at 75 μg/mL of CB NPs (184 ± 26% of levels in GnRHa-treated control cells, *p* < 0.05) and maintained at 100 μg/mL of CB NPs. In contrast, CB NPs did not modify the induction of *Lhb* transcript levels by GnRHa. In some experiments, GnRHa was replaced by activin A (10 ng/mL) following the same protocol. As expected, activin also significantly increased *Fshb* transcript levels (533 ± 16%, *p* < 0.05), however, CB NP treatment did not affect activin regulation unlike what was observed for GnRHa (Figure 8B).

## Discussion

Although there is now growing evidence that NPs affect the female reproductive system, the underlying mechanisms are still poorly understood (Iavicoli et al., 2013). Moreover, the detrimental effects of NPs on adult reproductive activity have been addressed mainly at the levels of the gonads and only little information is available on their possible impact on pituitary gonadotropins despite the crucial role of the latter in the maintenance of normal reproductive function. Here, we report that CB NPs increase FSH synthesis and release by pituitary gonadotrope cells both *in vivo* in female mice and *in vitro* in rat cultured pituitary cells, as well as in the gonadotrope cell line LβT2. In contrast, LH synthesis was unaffected or only marginally affected, highlighting the view that exposure to CB NPs may lead to a gonadotropin imbalance. Because the dose of CB NPs used in our study was occupationally relevant (Sanfins et al., 2011; Bourdon et al., 2013), our results underline the fact that exposure to CB NPs may be detrimental for female reproductive health.

The observed increase in FSH synthesis and release in female mice subjected to inhaled CB NP exposure was not associated with any significant changes in sex steroids or inhibin levels, suggesting that FSH increase is not the consequence of CB NP-induced alterations of the feedback control exerted by the gonads on pituitary activity. In contrast to FSH, no variations in circulating LH levels could be detected in CB NP-exposed females. Since numerous studies in animals have illustrated that pulsatile LH secretion closely reflects GnRH secretion by the hypothalamus, this result strongly suggests that CB NPs do not disrupt the activity of hypothalamic GnRH neurons. Altogether, these results indicate that, among the three endocrine organs of the reproductive axis – hypothalamus, pituitary and gonads – the pituitary could be particularly sensitive to CB NPs. A general effect of intranasal or intratracheal instillations reported for distinct types of NPs, including carbon NPs, is to induce a pulmonary inflammation at the site of deposition (Braakhuis et al., 2014). Since cytokines are known to affect pituitary endocrine activity (Haedo et al., 2009), such an inflammatory response could have contributed to the observed changes in gonadotropin secretion. LH synthesis and release have indeed been reported in numerous studies to be altered by cytokines, including IL-1β, IL-6 and TNFα, either *in vitro* (Russell et al., 2001; Haedo et al., 2009) or *in vivo* (Dondi et al., 1998; Makowski et al., 2020). Accordingly, the suppressive effect of inflammation induced by LPS or endotoxin on GnRH secretion has been documented in females from several species (Barabas et al., 2020). However, we did not observe any significant alteration of LH levels following exposure to CB NPs. Furthermore, even though the effect of pro-inflammatory cytokines on FSH synthesis and release in rodents has only been poorly addressed, IL-1β has been reported to decrease rather than increase FSH circulating levels in female rats (Dondi et al., 1998) or activin-stimulated FSH secretion in rat anterior pituitary cells (Bilezikjian et al., 1998). Since the pattern of the gonadotropin changes observed in the present study does not correspond with the changes reported to be induced by cytokines, it is unlikely that the enhancement of FSH secretion in female mice exposed to CB NPs is a consequence of an associated inflammatory response.

Recent studies have shown that the deposit of CB NPs into the lungs following NP entry into respiratory airways causes defects into organs that are distant from the NP deposition site. For example, inhalation of CB NPs during pregnancy damages cerebrovascular functions in female mice (Zhang et al., 2019) in addition to inducing neurodevelopmental changes in their offspring (Umezawa et al., 2018). Other studies have shown that intratracheal instillation of CB NPs causes genotoxicity in the liver of female mice (Modrzynska et al., 2018) and alters testosterone production and daily sperm production in testes of adult male mice (Yoshida et al., 2009). One possible mechanism explaining such changes would be a direct action of CB NPs on these organs. Many NPs are indeed reported to translocate from the lung into the blood circulation and this has been described in particular for intratracheally instilled CB NPs in mice (Shimada et al., 2006). Once in the blood, CB NPs can translocate into secondary organs as recently observed in the liver of mice (Modrzynska et al., 2018). As carbon NPs have been described to accumulate in organs without being effectively eliminated (Elgrabli et al., 2008; Czarny et al., 2014; Modrzynska et al., 2018), it is tempting to speculate that CB NPs may accumulate into the anterior pituitary gland and eventually alter pituitary endocrine activity *in vivo* as we have observed here in cultured pituitary cells. However, while it would have been interesting to address the issue of CB NP localization and accumulation *in vivo*, this is particularly difficult given the carbonaceous nature of the NPs of interest. Indeed, without labeling (which would in turn modify their physico-chemical characteristics and subsequent behavior), CB NPs cannot be visualized in a biological system since it is not possible to distinguish their chemical nature from the biological background signal (Boczkowski and Lanone, 2012).

Our finding that CB NPs differentially regulate the two gonadotropins *in vivo* is reinforced by our cell-based studies. Indeed, in both primary cultures of pituitary cells and gonadotrope LβT2 cells, CB NPs increase basal and GnRH-stimulated *Fshb* expression while leaving *Lhb* unaffected. Previous works using other types of CB NPs such as titanium dioxide (Gao et al., 2012) or nickel (Kong et al., 2014) NPs also reported effects on gonadotropin levels. However, in contrast to CB NPs, exposure to nickel NPs increased both gonadotropin levels while titanium dioxide NPs administration selectively decreased LH levels. The differences between these studies may be related to differences in the type of NPs, the mode of administration or the treatment duration. We have demonstrated in the present study that the cAMP/PKA pathway, which contributes to selective regulation of *Fshb* transcription by GnRH (Thompson et al., 2013), is rapidly recruited by CB NPs in LβT2 gonadotrope cells, as revealed by CREB phosphorylation. NPs, including CB NPs, have been shown to activate different signaling pathways such as MAPK, NF-kB or calcium pathways (Brown et al., 2000; Sydlik et al., 2006; Marano et al., 2011; Simon et al., 2017; Cheng et al., 2019) but this is the first demonstration, to our knowledge, of their ability to recruit the cAMP/PKA pathway. In our recent study using human granulosa cells, incubation with CB NPs under the same conditions as those used in this study rapidly activated the ERK1/2 signaling pathway (Simon et al., 2017) whereas it fails to stimulate the cAMP/PKA pathway (Simon V and Cohen-Tannoudji J, unpublished observation). These discrepancies observed between these two different cell types highlight the fact that NPs effects may depend not only on their shape, size and physicochemical properties but also on the cellular context. The recruitment of the cAMP pathway by CB NPs may be explained by a direct interaction of CB NPs with signaling entities regulating intracellular cAMP levels, including enzymes such as adenylyl cyclases or phosphodiesterases, or those located downstream of cAMP, such as PKA or its related transcription factor, CREB. Supporting this hypothesis is the demonstration, using the same CB NPs than those used in the present study, that CB NPs bind to the enzyme arylamine N-acetyltransferase, leading to changes in its protein conformational and enzymatic activity (Sanfins et al., 2011). It would be relevant in this context to study whether direct interaction of CB NPs with signaling entities of the cAMP pathway could occur in gonadotrope cells. Such a direct activation would explain why, despite being broadly distributed within gonadotrope cells, CB NPs could activate this signaling pathway. The blockade of the CB NP-mediated increase in *Fshb* expression by pharmacological inhibition of PKA in our experiment suggests that the recruitment of this pathway may be one of the mechanisms explaining the action of CB NPs on the expression of FSH. We observed that CB NPs amplified the effect of GnRH on *Fshb* but not *Lhb* gene expression in LβT2 gonadotrope cells. It would be of interest in future studies to assess whether GnRH regulation is also disrupted *in vivo*. In contrast to their effects on GnRH regulation, CB NPs did not affect the regulation exerted by activin, the major regulator of FSH. This could probably be explained by the inability of CB NPs to activate the Smad 2/3 signaling pathway, which is the main pathway mediating the action of activin on *Fshb* expression. Similarly, we observed no effect of CB NPs on the expression of the early growth factor protein-1 (Egr-1; not shown), a transcription factor key to controlling basal and GnRH-dependent *Lhb* transcription in gonadotrope cells (Halvorson et al., 1999; Wolfe and Call, 1999). This may explain, at least in part, the inability of CB NPs to increase *Lhb* expression.

FSH plays a crucial role during ovarian folliculogenesis, notably by promoting granulosa cell proliferation and estradiol synthesis (McGee et al., 1997; Robker and Richards, 1998). We did not, however, observe major alterations in the ovarian activity of female mice exposed to CB NPs as revealed by non-significant changes in circulating levels of sex steroids, and inhibin, or the pattern of ovarian cyclicity. It is possible that elevation of FSH was not maintained long enough to disrupt ovarian activity. *In vivo* exposure to CB NPs, at least during the period considered here, does not reproduce the effects observed previously when the human granulosa cell line KGN was directly exposed to CB NPs (Simon et al., 2017). There was, however, a trend toward a decrease in circulating estradiol levels in CB NP-treated mice. Analysis over longer periods of time would be necessary to further assess the potential consequences of CB NP-induced changes in FSH secretion on ovarian activity. In addition, the complex and diverse effects of CB NPs at the level of the whole organism, including the marked increase in FSH secretion reported here, may also have obscured the effect of CB NPs on the ovary. Interestingly, the absence of alterations in ovarian endocrine activity contrasts with alterations observed in testicular steroidogenesis and spermatogenesis in male mice intratracheally exposed to very similar-sized CB NP particles (14 vs 13 nm) (Yoshida et al., 2009). Although such discrepancies may be related the higher frequency of exposure of male mice to CB NPs compared to the exposure regimen in the present study, it may also reveal a differential susceptibility of gonads to CB NPs according to sex, as already reported in rats exposed to 2,3,7,8-tetrachlorodibenzo-pdioxin (Magre et al., 2012). Our demonstration, based on *in vitro* and *in vivo* studies, of a selective increase in FSH secretion in response to CB NP exposure, potentially underlines an alteration of the reproductive function as it is observed in patients or in animal models of ovarian insufficiency (Guigon et al., 2005; Vandormael-Pournin et al., 2015; Malini and Roy George, 2018). To summarize, our results show that CB NPs, by directly and/or indirectly altering the activity of anterior pituitary, could disrupt endocrine function in adult females, and consequently lead to adverse health effects. This reinforces the emerging idea that CB NPs, like other NPs, act as endocrine disruptors (Yoshida et al., 2009; WHO, 2012; Lu et al., 2013; Hutz et al., 2014; Simon et al., 2017). As human exposure to CB NPs is increasing worldwide, additional studies are needed to further assess the effects of such exposure on female fertility.

## Data Availability Statement

The original contributions presented in the study are included in the article/Supplementary Material, further inquiries can be directed to the corresponding authors.

## Ethics Statement

The animal study was reviewed and approved by ComEth Anses/ENVA/UPEC under the reference #12-104 (final approval #20/12/12-27).

## Author Contributions

CA: investigation, visualization, formal analysis, validation, data curation, and writing – original draft. EP, CD, and DL’H: investigation, data curation, and validation. GG: investigation, data curation, validation, and writing – review and editing. VG-M: investigation, visualization, validation, data curation, and writing – review and editing. RC: investigation and data curation. J-MD: funding acquisition and writing – review and editing. SL and JB: funding acquisition, writing – review and editing, and resources. VS: conceptualization, methodology, visualization, investigation, supervision, project administration, funding acquisition, and writing – original draft. JC-T: conceptualization, methodology, supervision, project administration, funding acquisition, and writing – original draft. All authors contributed to the article and approved the submitted version.

## Conflict of Interest

The authors declare that the research was conducted in the absence of any commercial or financial relationships that could be construed as a potential conflict of interest.

## Publisher’s Note

All claims expressed in this article are solely those of the authors and do not necessarily represent those of their affiliated organizations, or those of the publisher, the editors and the reviewers. Any product that may be evaluated in this article, or claim that may be made by its manufacturer, is not guaranteed or endorsed by the publisher.

## Abbreviations

FBS: fetal bovine serum
CB NPs: carbon black nanoparticles
Cga: glycoprotein hormone alpha polypeptide
CREB: cAMP response element-binding protein
ERK1/2: extracellular signal-regulated kinase 1 and 2
FSH: follicle-stimulating hormone
GnRH: gonadotropin-releasing hormone
HPRT: hypoxanthine phosphoribosyltransferase
IL-1 β: interleukin 1 beta
IL-6: Interleukin 6
JNK: Jun-kinase
LH: luteinizing hormone
MAPK: mitogen-activated protein kinase
MTT: 3-(4,5-dimethylthiazole-2-yl)-2,5-diphenyl tetrazolium bromide
NPs: nanoparticles
PKA: protein kinase A
PKC: protein Kinase C
TNF α: tumor necrosis factor alpha
SF3a1: splicing factor 3a subunit 1.
