# Supplementary figures and images for: Carbon Black Nanoparticles Selectively Alter Follicle-Stimulating Hormone Expression in vitro and in vivo in Female Mice
Source: Front Neurosci. 2021 Dec 6;15:780698. doi: 10.3389/fnins.2021.780698 (PMC8685435; doi:10.3389/fnins.2021.780698)

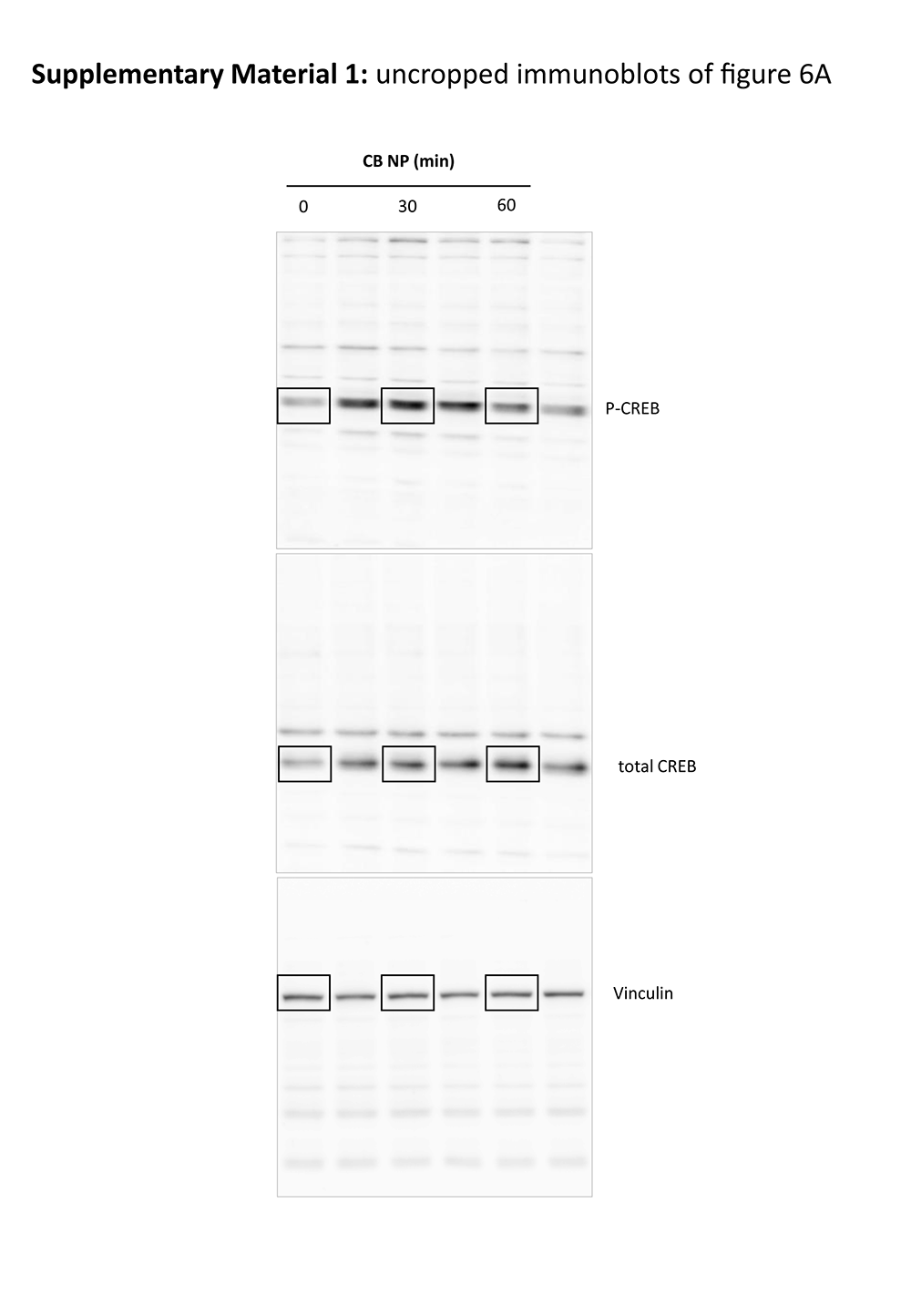

Supplement: Supplementary file 1 [file Image_1.TIF]

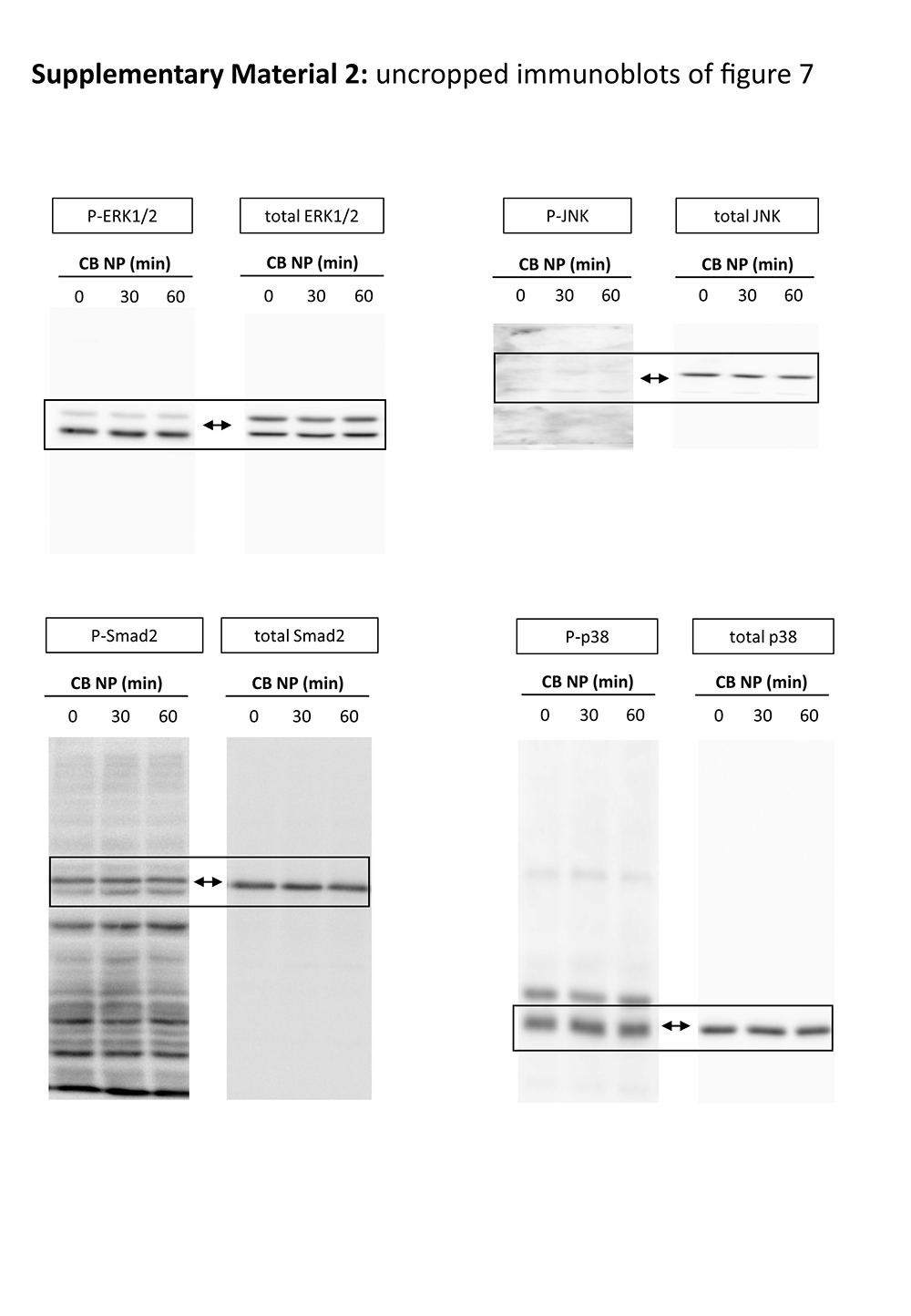

Supplement: Supplementary file 2 [file Image_2.TIF]
